# Implementation of strategies to prevent and treat postoperative delirium in the post-anesthesia caring unit: A German survey of current practice

**DOI:** 10.1007/s10877-020-00516-9

**Published:** 2020-05-09

**Authors:** Thomas Saller, Klaus F. Hofmann-Kiefer, Isabel Saller, Bernhard Zwissler, Vera von Dossow

**Affiliations:** 1 Department of Anaesthesiology, University Hospital, LMU Munich, Munich, Germany; 2 Department of Intercultural Communications, LMU Munich, Munich, Germany; 3 Institute for Anaesthesiology, Heart and Diabetes Center NRW, Ruhr University of Bochum, Georgstr. 11, 32545 Bad Oeynhausen, Germany

**Keywords:** Recovery room, Post-anesthesia nursing, Postoperative complications, Delirium, Health care survey

## Abstract

Postoperative delirium is associated with worse outcome. The aim of this study was to understand present strategies for delirium screening and therapy in German Post-Anesthesia-Caring-Units (PACU). We designed a German-wide web-based questionnaire which was sent to 922 chairmen of anesthesiologic departments and to 726 anesthetists working in ambulatory surgery. The response rate was 30% for hospital anesthesiologists. 10% (95%-confidence interval: 8–12) of the anesthesiologists applied a standardised screening for delirium. Even though not on a regular basis, in 44% (41–47) of the hospitals, a recommended and validated screening was used, the Nursing Delirium Screening Scale (NuDesc) or the Confusion Assessment Method for the Intensive Care Unit (CAM-ICU). If delirium was likely to occur, 46% (43–50) of the patients were examined using a delirium tool. 20% (17–23) of the patients were screened in intensive care units. For the treatment of delirium, alpha-2-agonists (83%, 80–85) were used most frequently for vegetative symptoms, benzodiazepines for anxiety in 71% (68–74), typical neuroleptics in 77% (71–82%) of patients with psychotic symptoms and in 20% (15–25) in patients with hypoactive delirium. 45% (39–51) of the respondents suggested no therapy for this entity. Monitoring of delirium is not established as a standard procedure in German PACUs. However, symptom-oriented therapy for postoperative delirium corresponds with current guidelines.

## Introduction

The recovery unit, also known as post-anesthesia caring unit (PACU), provides ongoing nursing and medical care by specially trained personnel until the patient has completely emerged from anesthesia. German guidelines postulate the permanent presence of an anesthesiologist. By all means, a trained anesthesiologist must be on short call [1].

### Delirium

Postoperative Delirium (POD) may occur, especially in the elderly, in up to 50% of the patients and is accountable for increased mortality [2]. A recent study showed that up to 19% of post-surgical patients developed delirium at the ward [3], while 14% were already tested positive for delirium in the PACU in the same setting [4]. Already 10 min after PACU admission, a pathologic RASS or NuDeSc-Score, often described as emergence delirium (ED), was associated with later delirium on the ward (OR 2.4; 1.5–3.9 CI) and death after 3 months (OR 1.4; 0.7–3.4 CI) [5]. Immediately after awakening and extubation, Monk found a 3.7% incidence of ED, declining to 1.3% when re-evaluated in the PACU [6]. Another study found a 4% incidence of ED at the time of discharge from the PACU [7]. In contrast to ED, hypoactive POD has subtle symptoms and is even more frequent. To detect POD in the PACU, objective tools, like pupillometry or processed electroencephalography were evaluated but are still not implemented into clinical practice [8, 9]. Today, a standardised delirium screening as proposed by Radtke et al. [4] and the recent European Guideline on delirium, published after this survey was performed [10], underlines the importance of screening for POD in all surgical patients. Screening should already start in the PACU and should be carried out in each shift up to postoperative day 5 with a validated score [10]. Besides the scientific studies that led to the development of the current guideline, there is no research or data outlining current strategies for POD management in the PACU. However, a detailed insight into the characteristics of clinical practice would be extremely fruitful for a successful future implementation of these guidelines [11].

To date, there are no data concerning organisational strategies and current practice in terms of delirium prophylaxis, use of delirium tools for screening and concerning delirium therapy in German PACUs. In order to achieve this information, we designed a prospective, German-wide online survey.

Our primary hypothesis was, that independently of the later recommendation to screen for delirium in the PACU, such a screening was not implemented at the time of the survey. Our secondary hypothesis was, that, nevertheless, medical prophylaxis and therapy of delirium is state of the art in German PACUs.

## Methods

This manuscript documents German standards in postoperative care via an online survey [12], meeting the COREQ criteria for the reporting of qualitative studies [13]. After reconciliation and approval by the ethics committee, Faculty of Medicine, LMU Munich, a mailing list provided by the German Society of Anaesthesiology and Intensive Care Medicine (DGAI) and the German Anaesthesiologists Association (BDA) was used to invite 922 heads of departments or anesthesiologists otherwise in an executive position for at least one of the 1173 heads of departments in German hospitals and 726 anesthesiologists working in an ambulatory setting to take part in the electronic survey (LimeSurvey 2.05 software package, LimeSurvey GmbH, Hamburg, Germany) by a single-use link sent by email. To remove responder bias, the questionnaire was re-sent once. In a short introduction we explained the significance of delirium monitoring in critical and postoperative care and the objective of the study. Informed consent was obtained before the 25 questions could be answered anonymously. All participation data, necessary for an email reminder, were erased after the completion of the survey.

### Statistical analysis

In order to estimate the relevant percent or proportion, results are being presented as correlation of the answers to the total cohort together with a 95%-confidence interval, calculated by the Clopper–Pearson method. For comparing means, the Mann–Whitney-U-Test was used. Categorical data were analysed by the Χ^2^-test or Fisher’s exact test, determining the level of significance as α = 5%. To show reach and frequencies of different recommendations for delirium screening, a Total Unduplicated Reach and Frequency (TURF) analysis [14, 15] was executed. TURF analyses are typically used for market research. They are useful to identify the optimal design in a combination of products to achieve a maximum of distribution (range) and sales (frequencies). Adopted on screening methods in medicine, a TURF analysis enables the examination of the total acceptance for a multi-component measure by a combination of separate subgroups. For statistical analysis we used SPSS Statistics for Macintosh 25 (IBM Corp., Armonk, NY, USA).

## Results

### Structural data

The survey was open for participation between 4th of May and 21st of June 2015. Participation was 30% among hospital anesthesiologists (n = 275/922) and 6% among ambulatory surgery anesthesiologists (n = 44/726). Due to the low response rate in this subgroup, results are displayed for the whole group of anesthesiologists. Five participants declined to participate or opted out. 292 completed the questionnaire. Questions only referring to PACU care were answered by 276 anesthesiologists. Of these responders, 70 [95%-confidence interval 65–76] % were head of their department, 18 [14–23] % senior consultants and 12 [8–16] % other physicians. 237 (86 [81–90] %) of the anesthesiologists worked at a hospital, 39 (14 [10–19] %) in an outpatient setting. 19 (7 [4–10] %) worked at a university hospital, 39 (13 [10–18] %) were associated with a university at a teaching hospital. Further structural data on the responders were published before [11], see also Table 1.

**Table 1:** Number of anesthetic procedures per year among the participants

Anesthetic procedures per year	Frequency	Percentage, ratio (%)
0–2500	43	14.7
2501–5000	67	22.9
5001–15,000	143	49.0
15,001–30,000	30	10.3
30,001–45,000	5	1.7
> 45,000	4	1.4
Total	292	100.0

### Organisational strategies regarding delirium in the PACU

In 19 [14–24] % of the hospitals, permanent medical care by a physician was exclusively provided for the PACU (Table 2). The continuous presence of an anesthesiologist was reported in > 75% only in hospitals with more than 30 PACU beds and more than 45,000 procedures per annum.

**Table 2:** Presence of an anesthesiologist in the PACU related to PACU capacity

Number of PACU beds	Physician presence in the PACU (n = 49), ratio (%)
1–4	26.5
5–9	13.8
10–19	16.5
20–29	33.3
> 30	75.0^a^

^a^Significant difference to other groups, p < 0.009

In 10 [6–14] % of the participating hospitals a structured delirium screening was being provided postoperatively in the PACU.

To detect a delirious state, 44% of the anesthesiologists who routinely score for delirium, used one of the two recommended tools at the PACU for in- as well as outpatient care: the Confusion Assessment Method for the Intensive Care Unit (CAM-ICU; 11 [8–16] %) and the Nursing Delirium Screening Scale (NuDESC 12 [8–17] %). 5% even had two tools in use (see Table 3). 65 [59–70] % of the respondents did not use any score at all. Patients diagnosed positively for delirium were mostly transferred to an intensive care unit (72 [66–77] %); 16 [12–20] % were transferred to a regular ward, despite being delirious. When delirium was diagnosed, 38% of the anesthesiologists in hospitals informed ward nurses about the delirium, 46% notified the physician in charge. To ensure individual postoperative care, 14 [10–19] % of the responders organised an individual bedside observation or monitor for the delirious patient (12 [8–17] %). In addition, 12 [8–17] % of the responders consulted a psychiatrist.

**Table 3:** Instruments used for delirium detection in the PACU

Screening instrument	Totals	Ratio (%)
CAM (Confusion Assessment Method)	10	3.6
CAM-ICU (CAM for the Intensive Care Unit)	27	9.7
DDS (Delirium Detection Scale)	7	2.5
DRS-R98 (Delirium Ratings Scale Revised 98)	0	–
ICDSC (Intensive Care Delirium Screening Checklist)	5	1.8
NuDesc (Nursing Delirium Screening Scale)	28	10.1
DSM (Diagnostic and Statistical Manual of Mental Disorders) or ICD-10	24	8.7
PAED (Pediatric Anesthesia Emergence Delirium Scale)	10	3.6

### Delirium screening at hospitals wards

**Fig. 1:**
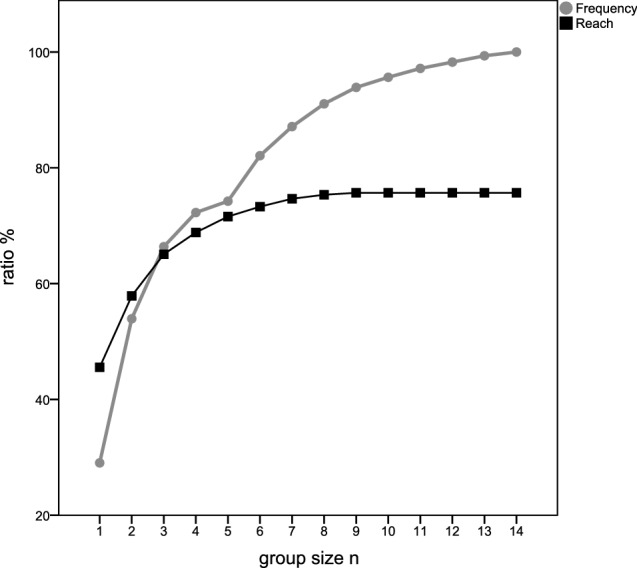
TURF-analysis. In a weighted TURF-analysis on delirium screening for the relevant groups, recommendations for delirium screening fit German anesthesiologists’ expectations in a maximum of 221 participants (75.7%), if there was a recommendation for one or more of the following settings: delirious or noticeable neurological patients, elderly or geriatric patients, for in-hospital patients, particular or critical care, only postoperatively. It reaches > 95% of the patients and > 90% if “elderly” and “all patients” are excluded in a hospital cohort (220 participants, 75.3%). The conjunction of the subgroups in the latter example shows, together with the graph, that even with a clear indication to establish screening for delirium for all subgroups as described in Table 4, 25% of the participants have not been reached by any recommendation, meaning they would not screen any patient. Even in the traditional fields of critical care or when delirium is suspected, 65.1% participiants would administer a score

In our survey at 77 [71–82] % of the wards in participating hospitals, a delirium screening is carried out for at least one clinical condition (see Table 4), meaning one fourth of the participants would not screen for delirium under any circumstances. Delirium screening was applied in only 46 [40–52] % of the patients even when delirium was suspected. A TURF analysis showed that a maximum of 75% of all patients would be tested for delirium (Fig. 1).

**Table 4:** Circumstances of delirium screening in general

When delirium screening is performed^a^	Totals (n = 292)	Ratio (%)
When delirium is suspected	133	45.5
As circumstances demand	114	39.0
Only in critically ill patients	57	19.5
Only when neurological abnormalities occur	36	12.3
Only postoperatively	27	9.2
Special care (e.g. stroke unit, chest pain unit)	23	7.9
Geriatric wards	18	6.2
ALL patients	8	3.1
Only in the elderly	8	2.7
On PACU discharge	7	2.4
Also in pediatric patients	5	1.7^b^
On PACU admission and discharge	5	1.7
Normal care (ward)	3	1.0
Emergency room	1	0.3

^a^Response to the question “In what framework do you usually administer a delirium score?”, multiple answers suitable

^b^Significant difference to all groups (p = 0.001)

### Pharmacological treatment of postoperative delirium in the PACU

Neuroleptics were the drugs used in at least every fifth case of PACU delirium and in up to 77 [71–82] % for patients with delusions (see Table 5), whereas atypical neuroleptics like quetiapin were rarely used in the PACU.

**Table 5:** Medication used for the therapy of delirium

Medication used according individual indication, ratio (%)	Vegetative Symptoms	Anxiety	Psychotic symptoms, hyperactivity	Hypoactive delirium
Typical neuroleptics	40.2	20.7	76.8	19.9
Atypical neuroleptics	4.3	3.6	15.6	13.8
Long-acting benzodiazepines	9.4	48.2	9.4	2.9
Short-acting benzodiazepines	22.8	40.6	19.2	6.5
Alpha-2 agonists	82.6	29.0	38.0	10.9
Beta-blocker	11.2	0.4	1.4	0
Propofol	14.5	10.5	11.6	1.1
Physostigmine	5.8	0.4	4.0	12.0
No medication	2.2	8.2	3.6	45.2

^a^Answers to the question "Which medication do you use for the therapy of delirium in the PACU by symptoms?”. Total ratio (%) of answers (multiple answers suitable) in all respondents

For anxiety, benzodiazepines were used in every second case. For vegetative symptoms caused by delirium, alpha-2-agonists were administered in 83 [76–87] % of all cases.

Physostigmine, the antidote for anticholinergic acting drugs (its use was not included in the questionnaire but was provided as a free-text answer for the therapy of PACU delirium) was applied specially to treat the hypoactive form of delirium by 12 [8–16] % of the respondents. However, almost every second anesthesiologist did not treat hypoactive delirium.

### Preoperative strategies regarding delirium

During preoperative evaluation, 43.1 [37.2–49.2] % of all respondents in our survey informed patients about the possibility of a postoperative delirium.

## Discussion

Our survey shows that delirium screening rates were low in German PACUs at least at the time, before German and European guidelines pleaded for postoperative delirium screening as a standard operating procedure for evidence-based practice. Several scientific studies (that had been published before 2015) providing evidence for high delirium rates in the PACUs [10] had not yet led to a cultural change in delirium management at that time.

**Fig. 2:**
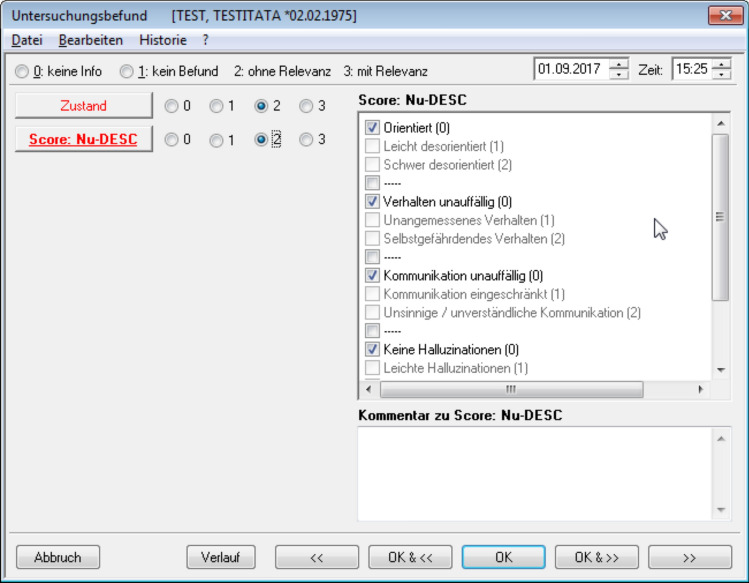
Example of delirium screening in electronic anesthesia documentation. The figure shows the interactive mask for the documentation of the NuDESC-delirium screening in the Narko Data^©^-monitor. University Hospital, LMU Munich

Only 10% of the participating hospitals screened for delirium in the PACU. 10% of the respondents had implemented structured programs at their facilities. 46% used a tool to screen patients at least if delirium was suspected and two third of the anesthesiologists did not use a delirium score in the PACU at all. In contrast, in an ICU-setting 72% of the anesthesiologists already used a scoring system [11], which leads to the conclusion that delirium screening in the ICU is much better established as it is in PACUs. It is reasonable to expect, that with further promotion of the recently published European Guidelines, an increase in screening rates will take place in the PACU as well. The implementation of a delirium screening test into electronic PACU documentation systems could further improve this (Fig. 2). In a study conducted at our center, we achieved delirium screening rates of more than 70% by implementing the Nurse Delirium Screening Scale requested by the staff at discharge from the PACU (unpublished data, submitted for publication).

To really realize our deficiencies concerning the screening for delirium—elaborated in the present research—could be a first step towards improving the patients’ worse outcome caused by delirium.

The second step should be to ensure the implementation of the guidelines. Therefore, information for all medical employees on delirium, e.g. with bedside teaching to the caregivers, is necessary. It has been documented that multicomponent programs for delirium prevention can reduce the incidence of delirium [16] and, in a geriatric population, the length of stay and hospital costs [17].

The introduction of an anesthetic protocol, designed to diminish adverse anesthetic effects, including delirium, is associated with a reduction of anesthetic recovery time [18] and thus reducing costs (about 10.80 € per minute for PACUs in a French study [19]). As a consequence, implementation of a structured protocol [20] for early delirium screening is cost-effective as it reduces PACU time and occupies fewer PACU nurses. A promising approach might be a standing order procedure, worked out by a multidisciplinary team of nurses, physicians and other experts, as the guidelines [10] suggest. A cue in the electronic documentation system could underline this order.

The third step would involve further raising awareness for the necessity of care for delirium among physicians as well as nurses and other caregivers. Our survey showed that in four out of five PACUs there is neither a physician permanently present nor did a structured delirium screening takes place (in 1 of 10 PACUs). Anesthesiologists on duty at the PACU—continuously as in university PACUs or on short call at others—could foster these considerations by acting as role models. Therefore, interdisciplinary communication between all personnel involved in delirium care would need to be improved as it has been identified as a major issue impeding successful guideline implementation [21].

### Delirium on the ward

Only every tenth hospital provided a structured screening for delirium at the time of the survey, 1% of the respondents practiced delirium screening in regular wards, 6% in geriatric wards and 10% in regular wards postoperatively. The severity and potential threat for patients is being recognised as 72% of delirious patients would be transferred to an intensive care unit for further care. If treatment on an ICU improves the non-pharmacological therapy of delirium is at least questionable.

Even if supposing that all patients at a hospital were screened, a maximum of 75% of the patients would be tested for delirium according to a TURF analysis [14] (Fig. 1). 25% of the caregivers would not be reached by any recommendation. Including these caregivers might be one of the biggest challenges when putting the recently published European Guidelines [10] into practice.

### Therapy of Delirium

Our study also showed that the therapy of delirium, once diagnosed, is very heterogeneous. The European Society of Anaesthesiology (ESA) recommendation for delirium therapy is to titrate haloperidol 0.25 mg-wise (level of recommendation ‘B’). Participants in our study administered haloperidol in 20–80% of the delirious patients, mostly according to their delirium subtype. Most of the anesthesiologists confirm a pathophysiological approach to the therapy of delirium. However, only half of the respondents treated hypoactive delirium, the subtype most frequent and at the same time most difficult to identify. Consequently, one has to assume that most of the delirious patients (especially with hypoactive delirium) were not correctly diagnosed and accordingly obtained no correspondent therapy. That finding implies on what future research might put a focus.

## Limitations

A qualitative online survey is a viable method for the identification of barriers to adherence to and implementation of guidelines [22]. In every qualitative study, however, a bias of social desirability must be assumed, thus possibly leading to more positive findings than a neutral observation of organizational practice could reveal. Since participation in an online survey can be carried out anonymously, false answers are negligible.

Neither surgeons nor nurses participated in the survey, thus, the results of this survey cannot be transferred to specialties other than anesthesiology. However, our work may overestimate the level of implementation by focusing on the expertise of leading physicians.

With 30% for hospital anesthesiologists, its participation rate lies within a typical range for online surveys [12, 23] and fits the response rate of the same cohort [24]. Our respondents represent a fifth of all leading German anesthesiologists.

Our data reflect the German perspective of delirium management before the implementation of the ESA guideline in 2017 [10]. Further studies should now examine whether the publication of the guideline has changed delirium management. Lastly, one would need to discuss, while taking into consideration the differences in health systems and intercultural issues, how these results might be transferable to other national contexts.

## Conclusion

The aim of this study was to understand organisational practices concerning PACU medical personnel and strategies for delirium screening and therapy in German PACUs in hospitals and ambulatory anesthesia facilities. Only 10% of the participating hospitals in our survey provided a structured delirium screening for their postoperative patients in the PACU. If already implemented, validated scores were used. The hypoactive form of delirium was rarely treated with any medication.

Our results show furthermore that standards for delirium prophylaxis and screening in the PACU were low before the publication of the ESA guidelines [10]. According to these evidence-based and consensus-based guidelines on postoperative delirium, patients should not leave the PACU without having been screened for POD and screening is recommended up to the 5th postoperative day [10].

Future studies should examine implementation rates at short intervals in order to reveal obstacles in applying these guidelines.

## Abbreviations

PACU: Post-Anesthesia Caring Unit
POD: Postoperative delirium
ED: Emergence delirium
RASS: Richmond Agitation Sedation Scale
NuDESC: Nursing Delirium Screening Scale
OR: Odds ratio
CI: Confidence interval
CAM-ICU: Confusion Assessment Method for the Intensive Care Unit
TURF: Total unduplicated reach and frequency analysis
ESA: European Society of Anaesthesiology
