# Correction to: Implementation of strategies to prevent and treat postoperative delirium in the post‑anesthesia caring unit: A German survey of current practice

**DOI:** 10.1007/s10877-021-00770-5

**Published:** 2021-10-19

**Authors:** Thomas Saller, Klaus F. Hofmann-Kiefer, Isabel Saller, Bernhard Zwissler, Vera von Dossow

**Affiliations:** 1 Department of Anaesthesiology, University Hospital, LMU Munich, Munich, Germany; 2 Department of Intercultural Communications, LMU Munich, Munich, Germany; 3 Institute for Anaesthesiology, Heart and Diabetes Center NRW, Ruhr University of Bochum, Georgstr. 11, 32545 Bad Oeynhausen, Germany

## Correction to: Journal of Clinical Monitoring and Computing (2021) 35:599–605 10.1007/s10877-020-00516-9

The article: “Implementation of strategies to prevent and treat postoperative delirium in the post-anesthesia caring unit”, written by Thomas Saller, Klaus F. Hofmann-Kiefer, Isabel Saller, Bernhard Zwissler and Vera von Dossow, was originally published electronically on the publisher’s internet portal on 9 May 2020 without open access. With the author(s)’ decision to opt for Open Choice the copyright of the article changed on 19 October 2021 to ©The Author(s) 2021 and the article is forthwith distributed under a Creative Commons Attribution 4.0 International License, which permits use, sharing, adaptation, distribution and reproduction in any medium or format, as long as you give appropriate credit to the original author(s) and the source, provide a link to the Creative Commons licence, and indicate if changes were made. The images or other third party material in this article are included in the article’s Creative Commons licence, unless indicated otherwise in a credit line to the material. If material is not included in the article’s Creative Commons licence and your intended use is not permitted by statutory regulation or exceeds the permitted use, you will need to obtain permission directly from the copyright holder. To view a copy of this licence, visit http://creativecommons.org/licenses/by/4.0/. The original article has been corrected.

